# Deaf children’s early sign language access: a guiding values brief

**DOI:** 10.1093/jdsade/enag002

**Published:** 2026-01-27

**Authors:** Elaine Gale, Amber J Martin

**Affiliations:** Department of Education, Hunter College of the City University of New York, New York, NY, United States; Department of Psychology, Hunter College of the City University of New York, New York, NY, United States

## Abstract

We propose five guiding values for early sign language access as guidelines for change in the design and practice of early intervention and education of Deaf children. Applying these guiding values will facilitate language-rich environments and reduce the harms associated with language deprivation experienced by many Deaf children. These guiding values are distinct yet interconnected and are applicable for professionals and families alike.

Deaf children are still arriving to kindergarten with language delays caused by early language deprivation, despite years of professionals calling attention to this issue. Language deprivation occurs when a child has insufficient access to community languages, preventing them from acquiring their first language on a typical timeline. Importantly, delays in language development are not the result of inabilities of the child but the result of obstructions to language access in the Deaf child’s environment. Thus, it can be prevented with well-structured early intervention systems. To address the core problem of language deprivation, this brief presents five guiding values that should underlie early intervention services for Deaf children. It extends the Family Centered Early Intervention guiding values to emphasize the need for language acquisition that involves early access to sign language to prevent language deprivation.

To highlight the urgent need for change by centering early intervention around sign language access, we provide these guiding values for early sign language to help families and professionals facilitate language-rich environments. This is important in order to reduce or eliminate the incidence of language deprivation and provide Deaf children a fair opportunity to begin school with a strong foundation for learning and development. We propose the following five guiding values along with recommendations for professionals and families to put into action to ensure Deaf children’s language development is supported in the structure of early intervention: (1) understand social systems, (2) adopt a human rights framework, (3) promote Deaf adult involvement, (4) practice linguistic carework, and (5) ensure early sign language access. These guiding values are distinct yet interconnected and are applicable for professionals and families alike.

## Understand social systems

Deaf children’s language development, like all children’s, should be understood as supported by multiple embedded and dynamically interacting global, national, local, and family systems rather than as a task that individual children must complete. Global structures impact national policies, national policies impact regional support available to build and sustain early intervention, and the organization of local public and private sectors impact families’ participation in or exclusion from early intervention.

For example, conducting newborn hearing screening requires training in a medical field and takes place in a medical context. Consequently, these first services for Deaf infants are embedded within medical systems. Naturally, their interventions are medically focused. But the primary problem arising from deafness is not a medical problem—it is language deprivation. In order to prevent language deprivation, support for sign language learning must be integrated into early intervention systems. This may be easier to integrate in early intervention organized within a system other than a medical system. How these systems are organized, function, and relate impacts the ability of those systems to provide sufficient language access. Language deprivation is the result of a failure of these systems to provide adequate language access, not a failure of the child.

Recommendations for families and professionals:

(1) Learn how the systems we work and live in function to support or undermine Deaf children’s sign language development.
(2) Recognize where we do and do not have power, and use our capacity to work toward changes that improve Deaf children’s sign language development.

## Adopt a human rights framework

Human rights, established by the United Nations, are fundamental rights for everyone. Deaf children’s linguistic human right to sign language is recognized in the United Nations Convention on the Rights of Persons with Disabilities (UNCRPD). The Convention explicitly defines sign languages as human languages (Article 2). It also endorses “facilitating the learning of sign language and the promotion of the linguistic identity of the deaf community,” (Article 24), and “accepting and facilitating the use of sign languages” (Article 21). The language rights of Deaf children with Deaf parents are typically respected with sign language as their primary language at home. However, Deaf children whose parents do not sign are often exposed to spoken language only, which may limit Deaf children’s full language rights.

Recommendations for families and professionals:

(1) Recognize that Deaf children’s linguistic human rights are respected through the inclusion of sign language.
(2) Implement human rights agreements in advocacy work for Deaf children’s early sign language access.

## Promote Deaf adult involvement

Deaf children benefit from Deaf Community Cultural Wealth (DCCW) in early intervention. DCCW is a framework that recognizes the unique strengths and assets of Deaf individuals. This is important because Deaf individuals support Deaf children as well as hearing families and professionals by sharing their lived experience, knowledge, and skills, which in turn facilitate early language access. Moreover, access to Deaf adults with early intervention and sign language qualifications is equally important. With accessible language models from Deaf professionals and adults, hearing families and professionals also gain additional knowledge and confidence needed to provide Deaf children with language-rich environments.

Recommendations for families and professionals:

(1) Incorporate DCCW in early intervention to support young Deaf children’s linguistic capital.
(2) Include Deaf adults who have early intervention and sign language qualifications.

## Practice linguistic carework

Linguistic carework is the effort invested by communication partners to ensure that each understands and is understood by the other. It is also described as a *willingness* to share the burden of communication, to create meaning, and ensure mutual understanding. It is context dependent, adjusting to the needs of the individuals at the moment.

Deaf people’s perceptiveness of understanding and being understood lead to unique linguistic carework practices that are both necessary and valued in Deaf communities. For example, Deaf people pause and wait for others’ visual attention before beginning or continuing a conversation. Deaf adults use linguistic carework practices with Deaf infants and children that can be adopted by families and professionals. It is an important part of early language development because it ensures access to both the meaning and form of language. Deaf children of all ages thrive with families and early intervention professionals who are committed to linguistic carework. Linguistic carework must become praxis in early intervention with Deaf children.

Recommendations for families and professionals:

(1) Be mindful of the ways behaviors either promote or hinder mutual understanding.
(2) Show flexibility and willingness to communicate by actively problem-solving communication barriers.

## Ensure early sign language access

Deaf children need consistent language exposure before age five to develop language competence and achieve equity in their educational and social experiences. This solid language foundation is crucial for a healthy quality of life, as it underpins cognitive development, social skills, and emotional well-being. When early language access is compromised, language deprivation poses serious health risks, impacting overall development. The best way to maximize early language access is to complete newborn hearing screening by one month of age, follow up by three months of age and immediately enroll in an early intervention program that includes deaf adults by six months of age. Additionally, tracking language milestones for both sign and spoken languages every six months is essential for families and professionals to monitor language progress and identify gaps early. This gives them time to adjust support so children receive the rich language access they need to be ready for kindergarten.

Recommendations for families and professionals:

(1) Complete newborn hearing screening by one month, follow up by three months, and enroll in an early intervention program that includes Deaf adults by six months.
(2) Track Deaf children’s sign language growth between birth and five years of age to ensure they are meeting language milestones and are kindergarten ready.

## Conclusion

This brief outlined five guiding values essential for ensuring early sign language access for Deaf children. The values underscore the interconnectedness between social systems, ethical frameworks, program structure, and Deaf children’s early language experiences. These values should underlie the development, organization, and implementation of early intervention for Deaf children and their families.
